# Rapid and sensitive detection of *Mycobacterium tuberculosis* using nested multi-enzyme isothermal rapid amplification in a single reaction

**DOI:** 10.1128/spectrum.00887-24

**Published:** 2024-10-28

**Authors:** Yingchao Chang, Mi Zhang, Gaowen Liu, Xinlin Wu, Qiaolu Yan, Cuixian Yang, Li Liu, Yue Feng, Xueshan Xia

**Affiliations:** 1 Faculty of Life Science and Technology, Kunming University of Science and Technology, Kunming, Yunnan, China; 2 Medical Laboratory, Yunnan Provincial Hospital of Infectious Disease, Kunming, Yunnan, China; 3 Yunnan Kecan Biotechnology Co., Ltd, Kunming, China; 4 Department of Respiratory and Critical Care Medicine, Dali Bai Autonomous Prefecture People's Hospital, Dali, Yunnan, China; 5 Yunnan Provincial Key Laboratory of Public Health and Biosafety, Kunming Medical University, Kunming, Yunnan, China; Quest Diagnostics Nichols Institute, Chantilly, Virginia, USA

**Keywords:** *Mycobacterium tuberculosis *(Mtb), nested multi-enzyme isothermal rapid amplification (nestMIRA), lateral flow dipsticks (LFD) strips

## Abstract

Tuberculosis (TB) remains a major global health problem, and there is an urgent need for rapid, sensitive, and easy-to-use diagnostic technologies to improve TB diagnosis. In this study, we developed the nested multi-enzyme isothermal rapid amplification (nestMIRA) assay for TB. We designed several pairs of primers and probes targeting the IS6110 sequence of *Mycobacterium tuberculosis* (Mtb) and performed combinatorial testing to optimize the performance of the TB nestMIRA assay. The reaction can be performed at a constant temperature of approximately 40°C and completed within 30 minutes in the same tube without opening the central cap. There has been no cross-reactivity with common *non-tuberculous mycobacteria* (NTB) and respiratory pathogens. The TB nestMIRA assay has a minimum detection limit of 5 copies/μL for H37Rv genomic DNA and a limit of quantification of 100 CFU/ml. To test the diagnostic performance of the TB nestMIRA assay, we conducted a 163-person clinical cohort study using comprehensive reference standards as the gold standard for clinical diagnosis. Our study showed that TB nestMIRA performed slightly better than GeneXpert MTB/RIF (Xpert) (85.27% vs. 82.17%) and significantly better than culture (55.81%) and acid-fast bacillus (AFB) smear (38.76%). The TB nestMIRA assay offers speed, specificity, sensitivity, and convenience. We believe that it has the potential to be a rapid alternative for TB diagnosis, particularly in resource-limited settings.

**IMPORTANCE:**

In this study, we have successfully developed a method called nested multi-enzyme isothermal rapid amplification (nestMIRA) for the detection of *Mycobacterium tuberculosis* (Mtb). This method involves a two-step thermostatic amplification process in the same tube and can be read using fluorescence and lateral flow dipstick (LFD) assays. It is known to be rapid, specific, and highly sensitive. Our method has shown promising results in the detection of clinical specimens, and we believe that it can be a valuable tool for the rapid detection of Mtb in a clinical setting.

## INTRODUCTION

Tuberculosis (TB) is a highly fatal disease caused by infection with the *Mycobacterium tuberculosis* (Mtb) complex. Historically, TB has been the leading cause of death among infectious diseases, with a higher mortality rate than AIDS and malaria worldwide (1). As reported by the World Health Organization, an estimated 10.6 million individuals worldwide fell ill with TB in 2022, representing an incidence rate of 133 cases per 100,000 population (1). Unfortunately, the COVID-19 pandemic has hampered efforts to prevent and control TB, causing a slowdown, stagnation, and even regression. This has deviated from the expected reduction rate needed to meet the established timeline of the End TB Strategy (1).

Early and accurate detection of Mtb is critical to the elimination of tuberculosis. TB diagnosis is primarily based on the composite reference standard (CRS), which includes clinical symptoms, radiological abnormalities, and laboratory tests (2, 3). However, acid-fast bacillus (AFB) smear and Mtb culture, which are routine laboratory tests, do not provide sufficient sensitivity to accurately diagnose TB, especially in children, people with HIV co-infection, and those with underlying metabolic diseases. GeneXpert MTB/RIF (Cepheid Inc, Sunnyvale, CA, USA) (“Xpert”) was developed in 2010 and approved by the World Health Organization for TB diagnosis (4). However, in 2021, only 38% of newly diagnosed TB patients had undergone Xpert testing (5). This is because the high cost of the test and its reliance on sophisticated equipment and specialized laboratories have limited its widespread use, particularly in remote and resource-poor areas. In addition, a recent study found that Xpert testing has not improved global detection rates and is not very effective in diagnosing extrapulmonary TB (6). Therefore, there is an urgent need to develop alternative methods for rapid screening and diagnosis of TB. Rapid and accurate diagnosis of TB is essential for effective treatment.

PCR-based assays, such as Xpert, are not suitable for point-of-care testing (POCT) for TB because they rely heavily on expensive thermal cyclers and heat-stable DNA polymerase. In contrast, isothermal amplification techniques such as recombinase polymerase amplification *(*RPA) (7), loop-mediated isothermal amplification (LAMP) (8), cross-priming amplification (CPA) (9, 10), recombinase-assisted amplification (RAA) (11), and multi-enzyme isothermal rapid amplification (MIRA) (12) have emerged as the new generation of preferred POCT methods for TB diagnosis. Both RPA and MIRA use recombinant enzymes to amplify complex DNA targets at room temperature in less than 15–30 minutes. MIRA incorporates a helicase that interacts with single-strand binding proteins (SSBs) to rapidly form the D-loop (13). In addition, MIRA uses a recombinant enzyme from *Streptomyces azureus* instead of T4 UvsX, resulting in improved amplification efficiency, stability, and resistance to interference compared to RPA (14).

These techniques are cost-effective and offer high specificity, sensitivity, and convenience, but these can face challenges such as false positives and insufficient signal amplification, especially when detecting low abundance clinical samples (7, 11). However, combining isothermal amplification with other signal amplification techniques can overcome these limitations (15, 16). The combination of nested isothermal amplification or Clustered Regularly Interspaced Short Palindromic Repeats (CRISPR) technologies has overcome the limitations of conventional PCR methods, resulting in significantly increased sensitivity (16, 17). However, there are concerns about the complexity and potential for cross-contamination of the developed semi-nested recombinase polymerase ampliﬁcation (snRPA) assay, which requires a biphasic protocol. On the other hand, a study successfully improved the sensitivity of an assay for *orientia tsutsugamushi* by snRPA, leading to the development of the snRPA assay (17). In this study, we developed an innovative assay for tuberculosis called TB nestMIRA that utilizes the attributes of nested MIRA reactions in a single tube. This method facilitates target gene detection by fluorescence and LFD assays.

## MATERIALS AND METHODS

### Preparation of DNA templates

In the study, distinct sample pre-processing protocols were meticulously formulated for varied clinical specimens. The sputum was admixed with a N-acetyl-L-cysteine (NALC)-NaOH solution in a 2:1 ratio and incubated at ambient temperature for 6 minutes, with intermittent vortexing and agitation two to three times for 5–10 s. Subsequent to incubation, 1 mL of the liquefied processed specimen was employed for DNA extraction. Homogeneous matrix specimens, including cerebrospinal fluid, thoracic and abdominal effusions, and blood, were directly amenable to 1 mL DNA extraction. Pertaining to urine specimens, 10 mL of urine underwent centrifugation at 1,500 rpm for 5 minutes to remove urinary sediments. Thereafter, centrifugation at 14,000 g for 10 minutes was conducted, and the resultant pellet was resuspended in 1 mL of PBS prior to DNA extraction.

For strains (H37Rv) isolated from solid cultures, select isolates were resuspended in 1 mL of PBS to facilitate DNA extraction. Throughout all specimen extractions, the EX-DNA Mtb kit (Tianlong, Xi'an) was utilized. Finally, 5 µL of supernatant was collected as a template for subsequent experiments. The DNA concentration of the isolated strains was determined utilizing the Qubit 4.0 (Thermo Fisher, USA).

### Design and synthesis of nestMIRA primers and probes

**Fig 1:**
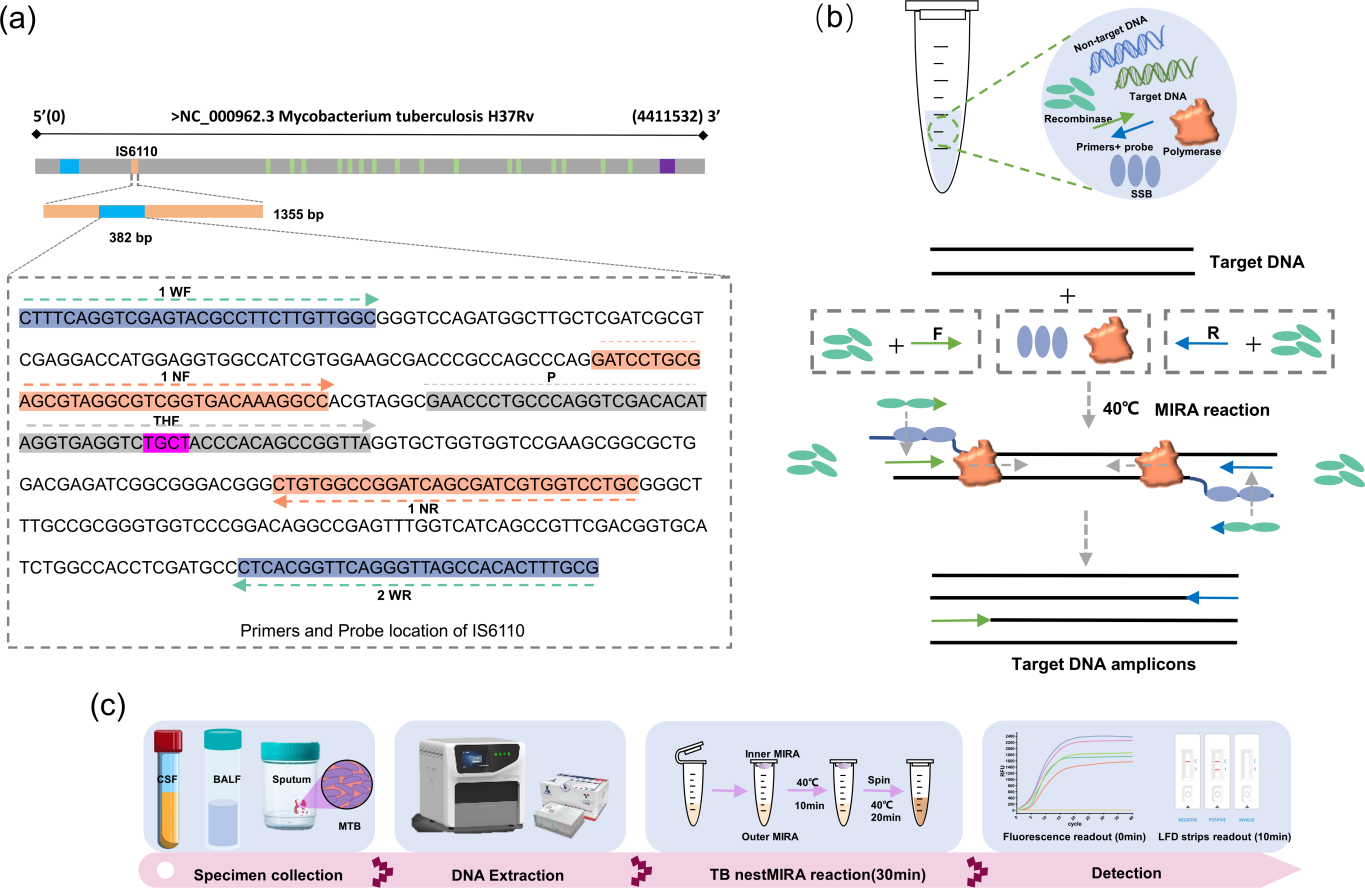
Schematic workflow of TB nestMIRA. (**a**) Positioning of optimal primer sets and probes: the study used the *Mycobacterium tuberculosis* IS6110 gene sequence, with optimal primer sets and probes strategically positioned. (**b**) MIRA operation: at 40°C, the recombinase-primer complex engages in base complementary pairing with homologous sequences in the genomic DNA, initiating strand exchange events. The displaced template strand binds to a single-strand binding protein (SSB), which prevents primer detachment during branch migration. The recombinase then dissociates, allowing DNA polymerase access to the 3′-end of the primer, initiating extension. This process is repeated, resulting in exponential DNA amplification. At the same time, the tetrahydrofuran (THF) site on the probe is specifically recognized by an endonuclease, generating a fluorescent signal or forming 5′-FAM,3′-Biotin double-stranded DNA. (**c**) TB nestMIRA workflow. TB nestMIRA consists of four sequential steps: sample collection, Mtb DNA extraction, nestMIRA reaction (20 min), LFD strip detection (10 min), or fluorescence readout (immediately displayed on a fluorescent screen).

For this study, a TB detection tool called nestMIRA was developed to detect multiple copies of a specific gene called IS6110 in the Mtb genome. This gene has an average of 13 copies in Mtb, making it a good target for detection. The primers and probes used in the study were designed to be specific for the IS6110 region, following the MIRA primer-probe design principles. The H37Rv strain of Mtb has 16 complete copies of IS6110. Primer-probe details and other relevant information are provided in Table S1 and Fig. 1a. Primers and probes were synthesized by gene synthesis (Taihe Biotechnology Co., Ltd, Beijing).

### Establishment and optimization of TB nestMIRA detection method

NestMIRA amplification of Mtb IS6110 was successfully performed in a single tube to establish the TB nestMIRA assay in this study. The TB nestMIRA assay includes a target nucleic acid template, an outer MIRA system, and an inner MIRA system. First, DNA was amplified in the outer MIRA using the DNA Isothermal Amplification Basic Kit (AMP-Future Biotech Co. Ltd, Weifang, China). DNA amplification and simultaneous fluorescence detection or LFD strip assays were then performed in the inner MIRA using the Fluorescence or Colloidal Gold DNA Isothermal Rapid Amplification Kit (AMP-Future Biotech Co. Ltd, Weifang, China). To perform the assay, we prepared both the outer MIRA system (25 µL) and the inner MIRA system (25 µL) according to the manufacturer's instructions. The outer MIRA system was loaded at the bottom of the tube and contained 14.7 µL A buffer, 1.0 µL outer primer set (10 µM), 5.0 µL DNA template, 2.05 µL ddH2O, and 1.25 µL B buffer (280 mM MgAc). On the other hand, the inner MIRA system was loaded on a lid containing 14.7 µL A buffer, 1.0 µL inner primer set (10 µM), 0.3 µL probe, 6.75 µL ddH2O, and 1.25 µL B buffer (280 mM MgAc).

TB nestMIRA assay is that both steps of thermostatic amplification are performed in the same tube without opening the cap in the middle. The pre-amplification step is performed in the cap of the tube at 40°C for 10 minutes. After centrifugation, the pre-amplified product is combined with the second step of the amplification system at 40°C for 20 minutes. Fluorescence detection is performed using a Gentier 96E (Tianlong, Xi'an, China) at a constant temperature with 30 second intervals between readings. For the Lateral Flow Strip assay, 10 µL of TB nestMIRA product is combined with 70 µL of ddH2O. Colloidal gold LFD strips are then added, and the mixture is incubated for 10 minutes. Results can be seen by eye or determined using a colloidal gold immunochromatographic analyzer.

### Evaluation of specificity and limit of detection

To validate the specificity of TB nestMIRA, we extracted genomic DNA from 12 NTM bacterial strains and six non-mycobacterial strains. These strains were provided by the Yunnan Infectious Disease Hospital and identified by Sanger sequencing. The NTM strains included *M. intracellulare, M. kansasii, M. abscessus, M. ulcerans, M. scrofulaceum, M. xenopi, M. chelonae, M. marinum, M. smegmatis, M. gordonae, M. avium*, and *M. fortuitum,* while the non-mycobacterial strains included *Klebsiella pneumoniae, A. baumannii, Proteus mirabilis, Rhodococcus equi, Staphylococcus aureus*, and *Escherichia coli*. The species specificity of TB nestMIRA was evaluated under optimized reaction conditions.

We evaluated the lower limit of detection (LOD) of H37Rv genomic DNA quantified colony-forming units (CFU). The copy number was calculated using the following formula: (6.02 × 10^23^) × (ng/μL × 10^−9^)/(DNA length × 660) = copies/μL. H37Rv genomic DNA was serially diluted 10-fold from 5 × 10^3^ copies/μL to 0.5 copies/μL and analyzed in triplicate. To measure CFU, e diluted a sample of viable bacteria from the reference strain H37Rv of Mtb and plated it on a solid culture medium. The colony-forming unit per milliliter was then determined from the number of colonies. Concentrations ranged from 500 CFU/mL to 25 CFU/ml and were then added to negative sputum samples. The TB nestMIRA assay was evaluated by determining the percentage of successful TB detections at different concentrations of H37Rv genomic DNA and each sputum input CFU concentration. A total of six replicates were conducted for each concentration sample. The LOD is defined as the minimum concentration at which 100% detection is consistently observed.

### Clinical study design and samples

A detailed study was conducted at the Yunnan Infectious Diseases Hospital from March to October 2023. The study included 226 patients with suspected active tuberculosis (TB), including both pulmonary and extrapulmonary manifestations. Ethical oversight was provided by the institution, and each patient or their designated representative gave informed consent according to established protocols. According to the Chinese National Standard for the Diagnosis of Pulmonary Tuberculosis (18) and the Clinical Treatment Guide for Tuberculosis in China (19), patients were categorized based on the combined clinical and laboratory diagnostic criteria as follows: (a) laboratory-confirmed TB or patients with bacteriologic confirmation of MTB (culture, AFB smear, GeneXpert MTB/RIF, positive by any method); (b) clinically diagnosed TB or patients with radiologic findings suggestive of TB and at least one of the following: clinical symptoms or signs of TB, positive tuberculin skin test (TST), or interferon-gamma release assay (IGRA), bronchoscopy, or histopathology consistent with TB; and (c) non-TB or patients diagnosed with other diseases who show improvement without anti-TB treatment. Specimens were accurately collected from designated sites of infection and immediately stored at −80°C to preserve sample integrity for subsequent diagnostic evaluations, including the TB nestMIRA assay. In this study, a double-blind design was employed, wherein the experimenters and outcome analysts were blinded to the TB status of the patients and the results of other diagnostic tests prior to the study.

### Data processing and statistical analysis

Statistical analyses were performed using MedCalc statistical software to determine the sensitivity, specificity, positive predictive value (PPV), negative predictive value (NPV), and kappa statistics of different diagnostic methods. Continuous variables were analyzed using the *t*-test, and categorical variables were analyzed using the chi-squared test. Results were expressed as ranges with 95% confidence intervals. Statistical calculations and graphs were performed using SPSS version 22.0, GraphPad Prism version 5, and web-based resources available at https://www.Chiplot.online.

## RESULT

### Establishment of IS6110-based TB nestMIRA assay

The TB nestMIRA assay is designed to be highly sensitive, simple, and user-friendly by nestMIRA to allow sequential amplification of target genes in a single tube (Fig. 1). The target sequence selected for this assay is the Mtb-specific insertion sequence IS6110, which is approximately 382 bp in length. Specific primers and probes were designed for this target sequence (Fig. 1a). In the nestMIRA reaction system, the Mtb DNA template first undergoes an outer MIRA reaction, facilitated by the functions of recombinase, single-stranded DNA binding protein, and DNA polymerase, at 40°C for 10 minutes. The template DNA is then further amplified in the inner MIRA reaction, while tetrahydrofuran (THF) is specifically recognized by nucleic acid exonuclease, resulting in cleavage of the probe. This process generates a fluorescent FAM signal or synthesizes double-stranded DNA (dsDNA) labeled with a FAM fluorophore at the 5′-end and biotin at the 3′-end. Ultimately, this assay allows the detection of the genomic presence of Mtb by fluorescence or LFD assays, confirming the presence of the target (Fig. 1b and c).

**Fig 2:**
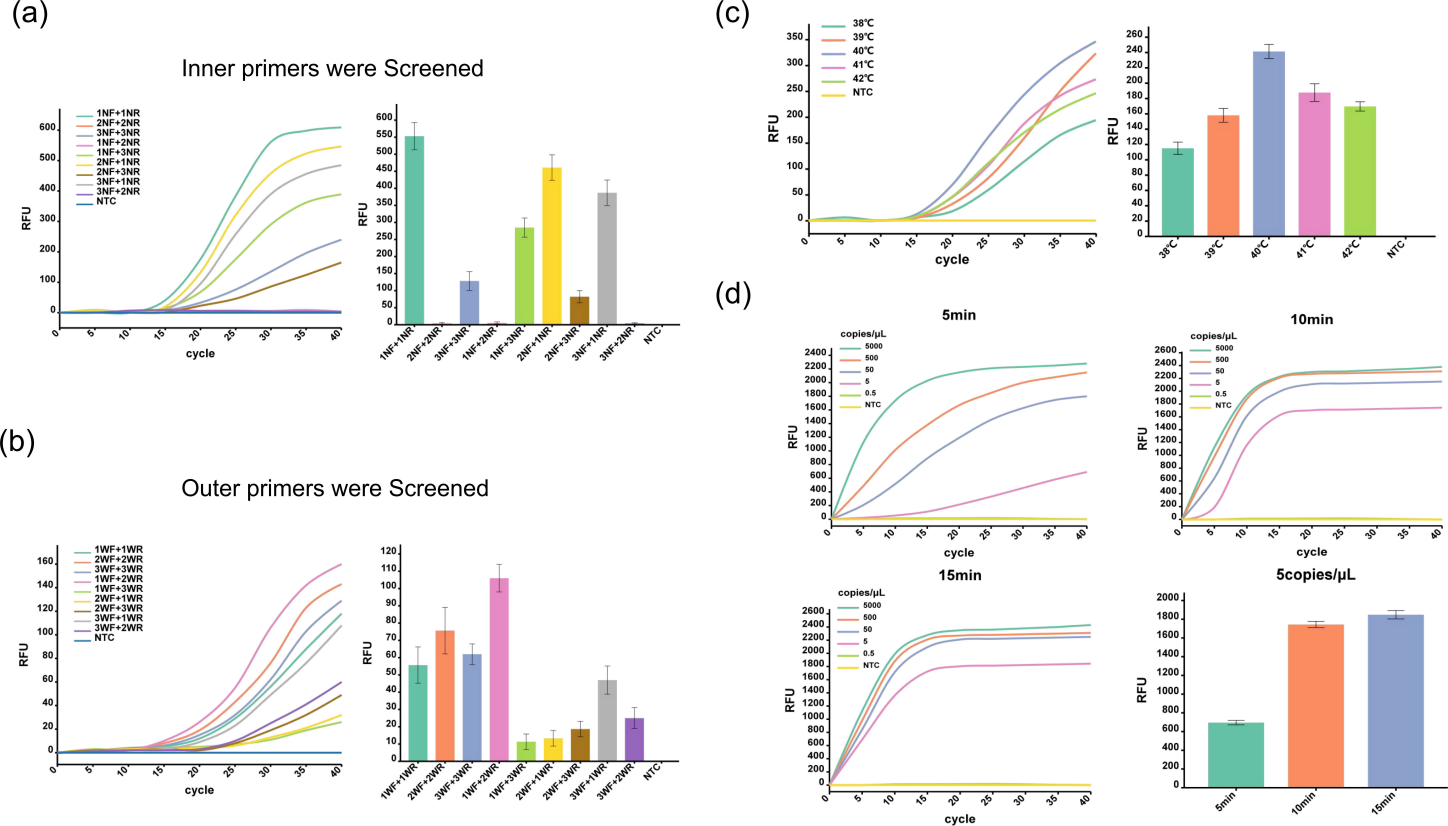
Establishment and optimization of the TB nestMIRA assay. (**a**) Inner primer combination screening: the study initiated screening for optimal peripheral primer combinations. (**b**) Peripheral primer combination screening: a comprehensive screening was performed to identify optimal peripheral primer combinations. (**c**) Reaction temperature optimization: to determine the ideal reaction temperature, we used a set of five temperatures ranging from 38°C to 42°C for optimization. (**d**) Optimization of TB nestMIRA outer MIRA preamplification time. Three different pre-amplification times (5 min, 10 min, and 15 min) were tested to optimize TB nestMIRA.

To identify the best primer sets for the TB nestMIRA assay, we designed three sets of outer primers and three sets of inner primers. We used H37Rv DNA at a concentration of 500 copies/μL as the template for the assay. We found that the 1NF + 1NR primer sets were the best for the inner primers (Fig. 2a) and the 1WF + 2WR primer sets were the best for the outer primers (Fig. 2b). After finalizing our primer sets, we evaluated five different reaction temperatures ranging from 38°C to 42°C (at 1°C intervals). We found that 40°C was the most effective reaction temperature (Fig. 2c). During the optimization process, we also realized that different pre-amplification times could cause variation in the detection time of positive results. To address this, we tested three different pre-amplification times: 5 minutes, 10 minutes, and 15 minutes. We found that as the pre-amplification time increased, the time to a positive result decreased. For pre-amplification times greater than 10 minutes, all samples (Mtb DNA concentration ranging from 5,000 copies/μL to 5 copies/μL) showed positive results within the first 5–15 minutes of the internal MIRA reaction (Fig. 2d). Consequently, the final preferred reaction time for the external MIRA was 10 minutes. With the optimized pre-amplification time, we were able to obtain a positive result in 30 minutes using TB nestMIRA.

### Specificity and sensitivity analysis of TB nestMIRA assay

**Fig 3:**
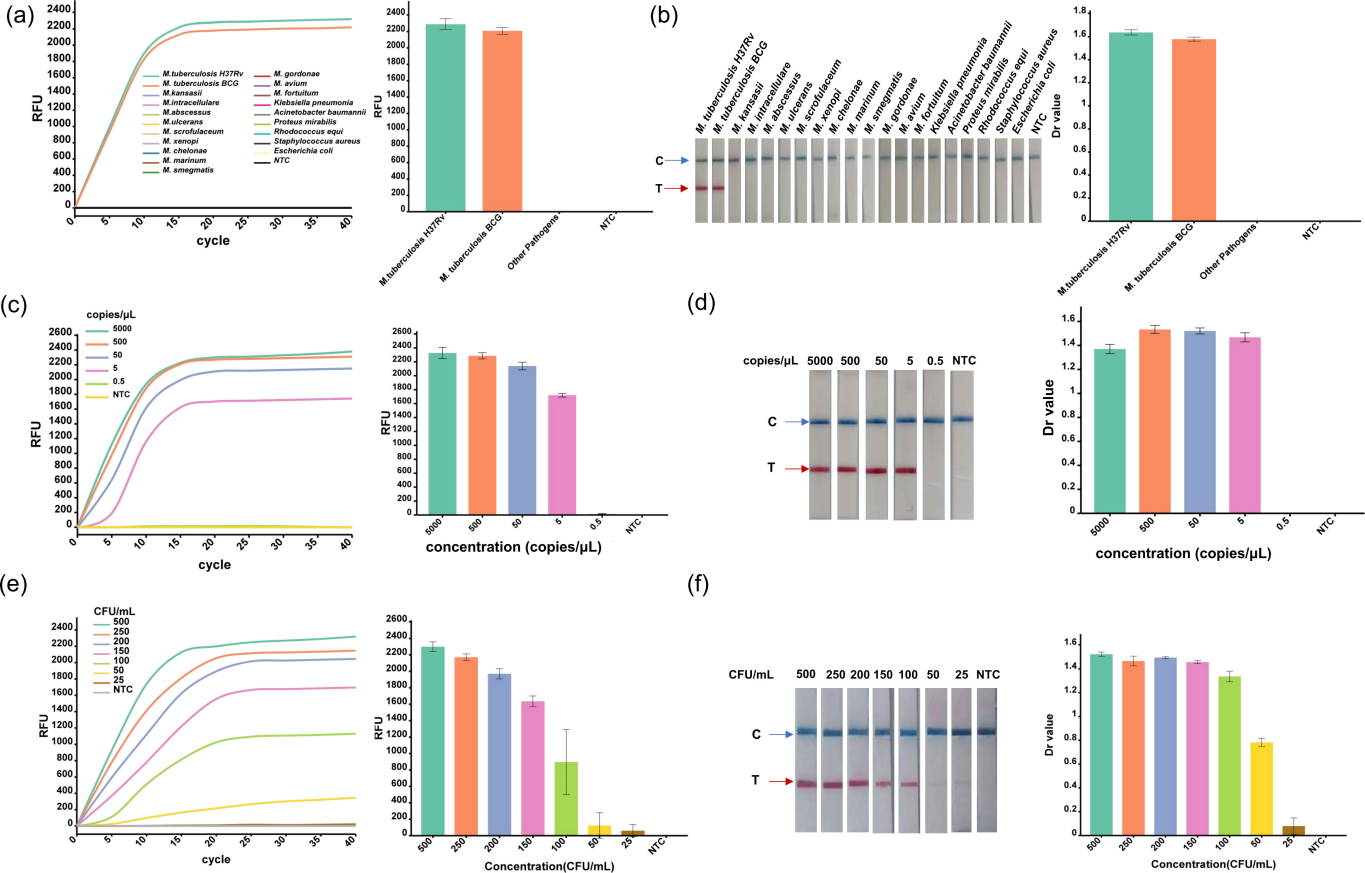
Specificity and sensitivity testing of TB nestMIRA. (**a and b**) Four common NTM strains (*M. intracellulare, M. kansasii, M. abscessus*, and *M. ulcerans*) and four non-mycobacterial strains (*Klebsiella pneumoniae, A. baumannii, Proteus mirabilis*, and *Rhodococcus equi*) were evaluated for specificity. (**c and d**) Serial dilutions of H37Rv genomic DNA at concentrations ranging from 5 × 10^3^ copies/μL to 0.5 copies/μL were performed for LOD. (**e and f**) Inactivated H37Rv strains at concentrations ranging from 500 CFU/mL to 25 CFU/ml were added to negative sputum samples and then subjected to the MIRA assay for tuberculosis nests to assess their LOD. Both fluorescence and LFD were used for detection and were plotted using the assay fluorescence values and Dr values [Dr = (T_Area)/(C_Area)].

The specificity of the TB nestMIRA assay was tested using genomic DNA from clinically characterized strains. Results showed that the assay did not detect fluorescence signals from 12 NTM bacterial strains and six non-mycobacterial strains. Only H37Rv and BCG strains showed higher fluorescence signals (>2,000) (Fig. 3a) and specific bands (Fig. 3b). There was no cross-reactivity with common non-tuberculous mycobacteria or respiratory pathogens. The TB nestMIRA assay was highly specific for these strains.

The study evaluated the LOD using two distinct nucleic acid targets. For the H37Rv genomic DNA, TB nestMIRA achieved a 100% positive detection rate when the concentration was equal to or greater than 5 copies/μL, thereby establishing a LOD of 5 copies/μL of genomic DNA (Fig. 3c and d), which is 10-fold higher compared to normal MIRA (Fig. S1). When TB nestMIRA was tested with simulated sputum samples containing H37Rv, a 100% positive detection rate was observed at concentrations equal to or greater than 100 CFU/mL (Fig. 3e and f). Consequently, the LOD for this method was definitively established at 100 CFU/mL.

### Patient characteristics

**Fig 4:**
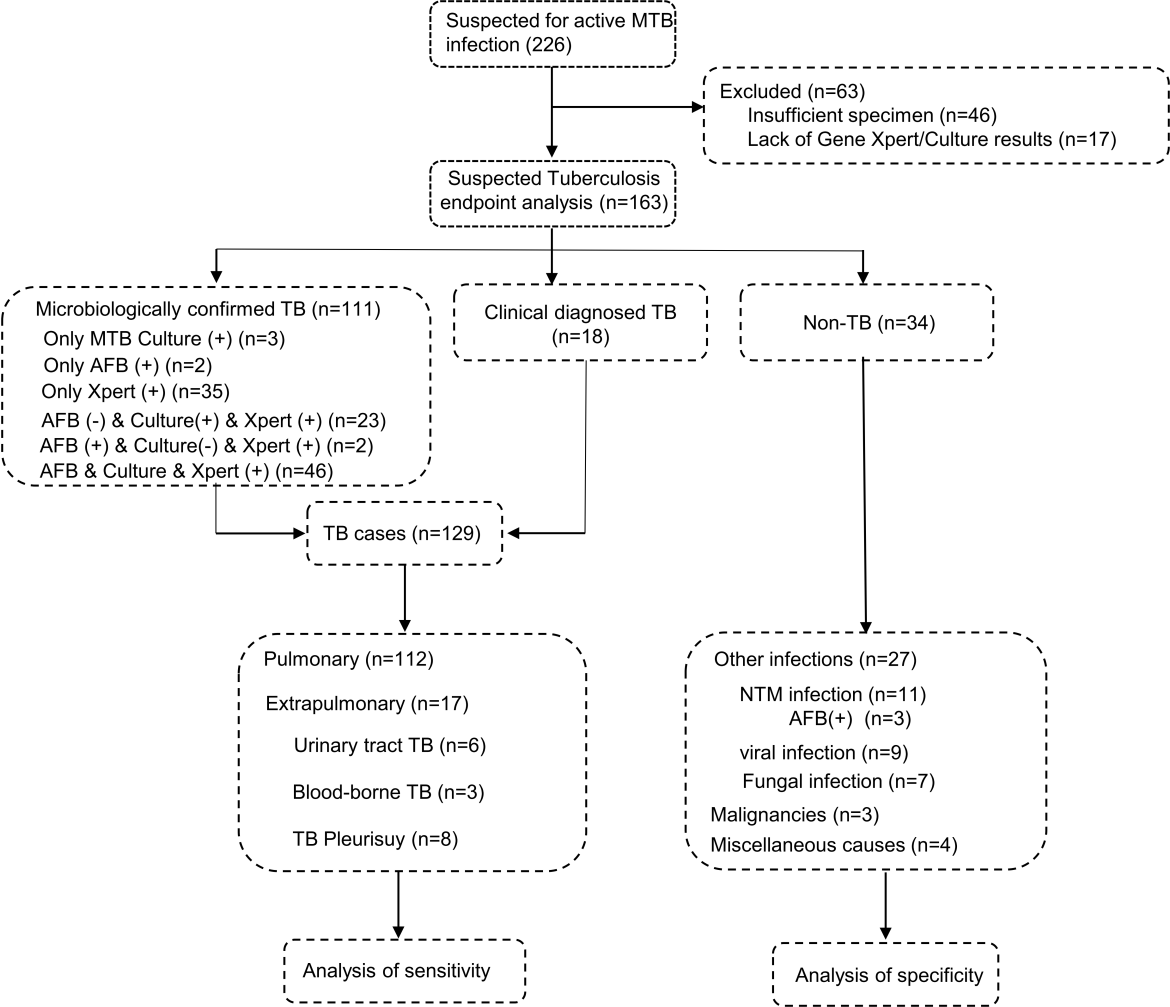
Recruitment and diagnostic classification of the participants.

A total of 226 sputum specimens were collected from patients suspected of having active tuberculosis. Of these, 63 cases were excluded due to uncertainty in the final diagnosis or insufficient specimen volume. The remaining 163 cases were tested for Mtb using various methods including AFB smear, culture, Xpert, and TB nestMIRA on direct clinical specimens. The clinicians divided the 163 cases into two groups based on the composite reference standard (CRS). The first group, consisting of 129 cases, was identified as the TB cohort, while the second group, consisting of 34 cases, was identified as the non-TB cohort (Fig. 4). Of the 129 cases in the TB cohort, 106 cases tested positive by Xpert MTB/RIF assay, 72 cases were confirmed positive by Mtb culture, and 50 cases were positive by AFB smear. In contrast, all 34 cases in the non-TB cohort were negative by both Xpert MTB/RIF assay and Mtb culture. It is important to note that, within the non-TB specimens, three AFB cases tested positive by AFB smear but were clinically diagnosed as NTM cases (Fig. 4).

Of the 163 participants, 129 were diagnosed with active Mtb infection. Among them, 112 cases (86.8%) were pulmonary tuberculosis and 17 cases (13.2%) were extrapulmonary tuberculosis. There was no significant difference in the age composition of the enrolled patients, but the proportion of males was higher (64.3% and 67.6% in the TB and non-TB groups, respectively). In addition, all participants were HIV-negative. More information on the characteristics of the enrolled patients is provided in Table S2.

### Application of TB nestMIRA in clinical tuberculosis

Table 1 shows the results of several tests performed on 129 confirmed TB cases in this study. The results showed that the TB nestMIRA test had a superior detection sensitivity of 85.27% compared to other tests such as the Xpert assay, culture, and AFB smear, which had detection sensitivities of 82.17%, 55.81%, and 38.76%, respectively. The kappa statistic method was then used to compare the detection results obtained by TB nestMIRA with those obtained by the other three methods (Table 1). The kappa coefficient for TB nestMIRA (0.707) was also higher than that for Xpert assay (0.658), culture (0.345), and AFB smear (0.164). Notably, there was no significant statistical difference in agreement between TB nestMIRA and Xpert assay (*P* > 0.05). However, TB nestMIRA had a significantly higher sensitivity and area under the receiver operating characteristic curve (AUC) than did culture and AFB smear (*P* < 0.05), as shown in Table 1 and Fig. S2.

**TABLE 1:** Diagnostic performance of several tests in confirmed cases of *Mycobacterium tuberculosis^a^^,^ ^b^*

Test	CRS diagnosis		Sensitivity% (95% Cl)	Specifivity% (95% Cl)	PPV% (95% Cl)	NPV% (95% Cl)	kappa value (95% Cl)	AUC (95% Cl）
	TB (*n* = 129)	Non-TB (*n* = 34)						
TB nestMIRA								
Positive	110	0	85.27 (77.96–90.89)	100.00 (89.72–100.00)	100.00 (96.70–100.00)	64.15 (49.80–76.86)	0.707 (0.589–0.826)	0.996 (0.989–1.000)
Negative	19	34						
Xpert MTB/RIF								
Positive	106	0	82.17^a^ (74.46–88.35)	100.00 ^a^ (89.72–100.00)	100.00^a^ (96.58–100.00)	59.65^a^ (45.82–72.44)	0.658^a^ (0.536–0.780)	0.911^b^ (0.868–0.954)
Negative	23	34						
Culture								
Positive	72	0	55.81^b^ (46.81–64.55)	100.00 ^a^ (89.72–100.00)	100.00 ^a^ (95.01–100.00)	37.36^b^ ^(^27.44-48.13)	0.345^b^ (0.242–0.449)	0.779^b^ (0.710–0.849)
Negative	57	34						
AFB								
Positive	50	3	38.76^b^ (31.31–47.73)	91.18^b^ (76.32–98.14)	94.34^b^ (84.34–98.82)	28.18^b^ (20.02–37.56)	0.164^b^ (0.0796–0.249)	0.655^b^ (0.564–0.746)
Negative	79	31						

^*a*^ Notes: “a” means that there is no statistically significant difference (*P* > 0.05); “b” means that there is a statistically significant difference (*P* < 0.05); CI, confidence interval; PPV, positive predictive value; NPV, negative predictive value; AUC, area under the curve; CRS, composite reference standard.

^*b*^ The Wilcoxon-Mann-Whitney statistical test was used to obtain the AUC, and the differences between the assays were compared by calculating the Z statistic, which was calculated as ，$Z=\frac{\left(p1-p2\right)}{\sqrt{\left(SE1^{2}+SE2^{2}\right)}}$where, SE = $\sqrt{p\left(1-p\right)/n}$. |Z| > 1.96 (corresponding to a significance level of α = 0.05), there is a significant difference.

**Fig 5:**
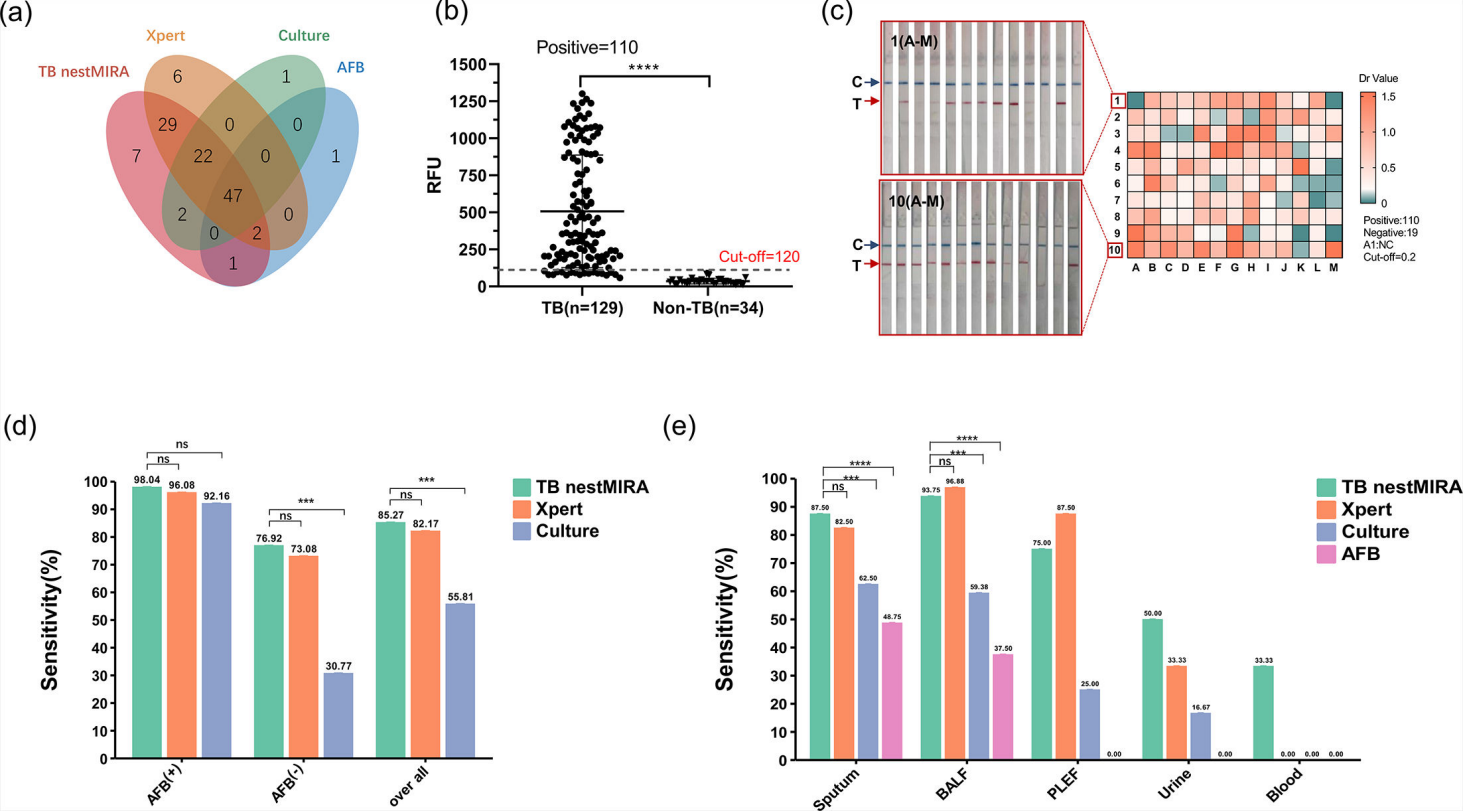
Performance of TB nestMIRA in clinical TB specimen testing. (**a**) Venn diagram of TB nestMIRA, Xpert, Mtb culture, and AFB smear results in TB specimens. (**b**) Inclusion of 163 suspected TB patients with active TB (*n* = 129) and non-TB patients (*n* = 34) tested for Mtb DNA. The dotted line represents the cut-off values at 120, and the black bars represent the mean and standard error of the mean (SEM). Unpaired *t*-test, ****P < 0.0001. (**c**) Results of LFD testing in 129 patients with active TB. larger Dr values (Dr = T_Area/C_Area) and darker colors indicate higher Mtb DNA loads in the samples. The first and 10th columns are representative LFD strip results. (**d**) Comparison of TB nestMIRA, Xpert, and Mtb culture results in AFB smear-positive and AFB smear-negative patients. Chi-squared test, ***P < 0.001, ****P < 0.0001. (**e**) Sensitivity of TB nestMIRA, Xpert, Mtb culture, and AFB smear in different clinical specimen tests. χ test, ***P < 0.001, ****P < 0.0001.

Samples were collected from 129 patients with tuberculosis and analyzed using multiple diagnostic methods, including AFB smear, culture, Xpert, and TB nestMIRA. The results were presented using a Venn diagram showing the overlapping distribution of these diagnostic methods. The number of positive samples identified through the MIRA-only approach was seven, in contrast to the six, one, and one positive samples identified through the Xpert-only, culture-only, and AFB-only methods, respectively (Fig. 5a; Table S3). Furthermore, when fluorescence and LFD strips were used to assess TB and non-TB cases, the fluorescence values were significantly higher in the TB group compared to the non-TB group (*P* < 0.0001) (Fig. 5b). In addition, the LFD strips showed a distinct positive band on the detection line (T-line) (Fig. 5c).

We conducted a study to evaluate the detection performance of three tuberculosis (TB) diagnostic tests, namely, TB nestMIRA, Xpert, and culture, in patients with both positive and negative AFB smear results. The study found that, in patients with a positive AFB smear, the detection rates of TB nestMIRA, Xpert, and Mtb culture were similar at 98.04%, 96.08%, and 92.16%, respectively (*P* > 0.05). In patients with a negative AFB smear, TB nestMIRA had a slightly higher detection rate of 76.92% compared to Xpert (73.08%), but the difference was not statistically significant (*P* > 0.05). However, the study showed that the detection rate of TB nestMIRA was significantly higher (*P* < 0.05) compared to culture (30.77%) (Fig. 5d).

We performed a comprehensive analysis of the diagnostic performance of the three methods in different sample types. We observed differences in test performance in different body fluids. In general, TB nestMIRA showed a higher positivity rate than Xpert, culture, and AFB smear, except in alveolar lavage fluid. TB nestMIRA was found to be suitable for the detection of extrapulmonary TB specimens such as urine, blood, chest, and abdominal fluids, showing a significantly higher positivity rate compared to the other three assays (Fig. 5e).

## DISCUSSION

Bacteria-based tests currently used for TB detection, such as AFB smears and Mtb cultures, have low sensitivity and are turnaround time (20, 21). This has led to a demand for significant improvements in TB diagnostics, including the need for decentralized POCT to complement centralized laboratory testing (22). Researchers have investigated the use of LAMP, RPA, and MIRA for TB detection (7, 11, 13, 23–25). However, these studies have mainly focused on a single amplification technique, often combined with Hydroxy-Naphthol Blue or LFD for visual Mtb detection. Less emphasis has been placed on method sensitivity and turnaround time. As a result, these approaches lack the sensitivity needed to detect low-abundance clinical samples, and the lack of clinical cohort studies raises concerns about their performance in clinical detection.

We conducted a study to improve the nestMIRA amplification. The result is the TB nestMIRA diagnostic platform. This platform uses both a fluorescent probe and dual primer sets to amplify and detect the IS6110 sequence in a single reaction tube. Compared to the snRPA assay, TB nestMIRA is more convenient and eliminates the risk of contamination (17). In addition, TB nestMIRA has an impressive LOD of 5 copies/μL for H37Rv genomic DNA and does not cross-react with non-tuberculous mycobacteria (NTM) strains. This assay is more sensitive than the previous RAA assay based on the IS1081 gene, which had an LOD of 163 copies/reaction (11). Furthermore, when compared to the LOD for three commonly used clinical TB tests—TB culture (10–100 CFU/mL) (26), AFB smear (500–10,000 CFU/mL) (27, 28), and Xpert (112.6–131.0 CFU/mL) (29)—our established method has a TB LOD of at least 100 CFU/mL (Fig. 3e and f), which is very close to the sensitivity of the Xpert method. These results demonstrate the high sensitivity of TB nestMIRA.

To evaluate the specificity of this method, we selected 12 clinical NTM (*M. intracellulare, M. kansasii, M. abscessus, M. ulcerans, M. scrofulaceum, M. xenopi, M. chelonae, M. marinum, M. smegmatis, M. gordonae, M. avium*, and *M. fortuitum*) for comparison with Mtb based on their taxonomic, pathogenic, and epidemiological similarities. Subsequently, the two most prevalent respiratory pathogens, *Klebsiella pneumoniae* and *A. baumannii*, were selected as were two common pathogens of other *Mycobacterium* spp (*Proteus mirabilis* and *Rhodococcus equi*) and the two most common clinical pathogens (*Staphylococcus aureus* and *Escherichia coli*). A total of 18 bacteria were used as samples to assess the cross-reactivity of the method. The results of the TB nestMIRA analysis showed that only Mtb samples tested positive, indicating that the method has good specificity.

Similar to nested PCR, the nestMIRA assay demonstrates high sensitivity. This is due to the thermostatic pre-amplification of the outer primers, which increases the number of templates, facilitating efficient amplification and robust fluorescent signal release from the inner primers and probe. The key feature of this method is that both steps of thermostatic amplification are performed in the same tube without opening the cap in the middle (Fig. 1c). While other CRISPR-based methods for TB detection have been established, they typically require a two-step process involving nucleic acid amplification followed by CRISPR-guided sequence-specific recognition (30, 31).

A clinical cohort study was conducted to evaluate the performance of TB nestMIRA in detecting clinical specimens. The study included 163 patients with suspected TB. The results showed that TB nestMIRA had a higher sensitivity (85.27%) compared to other diagnostic techniques such as Xpert (82.17%), culture (55.81%), and AFB smear (38.76%) (Table 1). Notably, TB nestMIRA outperformed culture (30.77%) and Xpert (73.08%) in detecting AFB smear-negative TB patients (Fig. 5d). This demonstrates that TB nestMIRA is a viable alternative to conventional diagnostic techniques (32). The diagnostic sensitivity of TB nestMIRA (85.27%) was within the range reported by previous CRISPR-based TB research (from 72.19% to 86.8%) (9, 31, 33, 34), although its LOD was not as low as some CRISPR-based TB studies. Differences in LOD assays, Mtb DNA extraction methods, and population heterogeneity in study cohorts may explain the observed variance among studies. Several clinical cohort studies did not adhere to the TB diagnostic composite reference standard, resulting in the development of assays with high sensitivities that did not match the sensitivities observed in real-world clinical settings (32, 35, 36). In our study, however, we established a representative cohort, and the detection sensitivities of TB nestMIRA (85.27%) and Xpert (82.17%) accurately reflected their clinical performance. The diagnostic results of the CRS (AUC = 0.996 for TB nestMIRA and AUC = 0.911 for Xpert) were in close agreement with our results (Table 1).

Unfortunately, the study conducted did not include a sufficient sample size of extrapulmonary TB cases. While the study yielded positive test results, additional research is needed to determine the efficacy of the assay in non-sputum specimens such as urine, blood, pleural effusion, and ascitic fluid. The traditional Mtb DNA extraction method was used in this study, and the development of a rapid Mtb extraction protocol remains a significant challenge. Although rapid nucleic acid release agents have proven effective for SARS-CoV-2 detection (37, 38), their application to Mtb is less effective due to the robust cell wall structure of Mtb and the complexity of clinical samples (39). Currently, sample pre-processing and nucleic acid extraction remain significant bottlenecks in the clinical translation of TB nestMIRA and CRISPR-based POCT (39). In the future, a streamlined workflow that accelerates the progression from sample introduction to result dissemination is envisioned, with quantifiable smart terminal output and immediate patient result feedback. This approach is expected to strengthen TB control initiatives and telemedicine interventions, which are goals we are passionately pursuing.

### Conclusion

In conclusion, our study create a TB nestMIRA assay that is characterized by its rapidity, high sensitivity, ease of use, and cost-effectiveness. This assay shows promise as a viable alternative for the molecular diagnosis of tuberculosis, especially in resource-limited settings.

## SUPPLEMENTAL MATERIAL

Supplemental figures spectrum.00887-24-s0001.docx Fig. S1 and S2.

Supplemental tables spectrum.00887-24-s0002.docx Tables S1 to S3.

ASM does not own the copyrights to Supplemental Material that may be linked to, or accessed through, an article. The authors have granted ASM a non-exclusive, world-wide license to publish the Supplemental Material files. Please contact the corresponding author directly for reuse.

## References

[1] World Health Organization. 2023. Global tuberculosis report 2023. https://reliefweb.int/report/world/global-tuberculosis-report-2023.

[2] Chaudhary J, Chawla DS, Gupta V, Singh A, Aggarwal M. 2023. Diagnostic efficacy of new Xpert ultra for extrapulmonary tuberculosis using culture and composite reference standard. Int J Appl Basic Med Res 13:224–229. doi:10.4103/ijabmr.ijabmr_348_23 38229732 PMC10789468

[3] Saxena R, Shrinet K, Rai SN, Singh K, Jain S, Jain S, Singh D, Anupurba S, Jain M. 2022. Diagnosis of genital tuberculosis in infertile women by using the composite reference standard. Dis Markers 2022:8078639. doi:10.1155/2022/8078639 36016849 PMC9398877

[4] Anonymous. WHO. 2011. Policy statement: automated real-time nucleic acid amplification technology for rapid and simultaneous detection of tuberculosis and rifampicin resistance: Xpert MTB/RIF system. Department/Division 26158191

[5] World Health Organization. 2022. Global tuberculosis report 2022. https://reliefweb.int/report/world/global-tuberculosis-report-2022.

[6] Huang Z, LaCourse SM, Kay AW, Stern J, Escudero JN, Youngquist BM, Zheng W, Vambe D, Dlamini M, Mtetwa G, Cranmer LM, Njuguna I, Wamalwa DC, Maleche-Obimbo E, Catanzaro DG, Lyon CJ, John-Stewart G, DiNardo A, Mandalakas AM, Ning B, Hu TY. 2022. CRISPR detection of circulating cell-free Mycobacterium tuberculosis DNA in adults and children, including children with HIV: a molecular diagnostics study. Lancet Microbe 3:e482–e492. doi:10.1016/S2666-5247(22)00087-8 35659882 PMC9300929

[7] Xu Y, Wu P, Zhang H, Li J. 2021. Rapid detection of Mycobacterium tuberculosis based on antigen 85B via real-time recombinase polymerase amplification. Lett Appl Microbiol 72:106–112. doi:10.1111/lam.13364 32726877

[8] Moon SH, Kim EJ, Tomono J, Miyamoto S, Mitarai S, Kim DW, Seki M. 2015. Detection of Mycobacterium tuberculosis complex in sputum specimens using a loop-mediated isothermal amplification assay in Korea. J Med Microbiol 64:1335–1340. doi:10.1099/jmm.0.000164 26338293

[9] Zhang Z, Du J, Liu T, Wang F, Jia J, Dong L, Zhao L, Xue Y, Jiang G, Yu X, Huang H. 2021. EasyNAT MTC assay: a simple, rapid, and low-cost cross-priming amplification method for the detection of Mycobacterium tuberculosis suitable for point-of-care testing. Emerg Microbes Infect 10:1530–1535. doi:10.1080/22221751.2021.1959271 34288833 PMC8330774

[10] Fang R, Li X, Hu L, You Q, Li J, Wu J, Xu P, Zhong H, Luo Y, Mei J, Gao Q. 2009. Cross-priming amplification for rapid detection of Mycobacterium tuberculosis in sputum specimens. J Clin Microbiol 47:845–847. doi:10.1128/JCM.01528-08 19116359 PMC2650920

[11] Liu Y, Ren W, Xue Z, Miao Y, Wang W, Zhang X, Yao C, Shang Y, Li S, Mi F, Pang Y. 2023. Real-time recombinase-aided amplification assay for rapid amplification of the IS1081 gene of Mycobacterium tuberculosis. Eur J Clin Microbiol Infect Dis 42:963–972. doi:10.1007/s10096-023-04626-5 37256455

[12] Li M, Lu Y, Liu H, Lin S, Qian C, Nan X, Li G, Zhao X, Wan K-L, Zhao L. 2023. Rapid detection of fluoroquinolone resistance in Mycobacterium tuberculosis using a novel multienzyme isothermal rapid assay. J Antibiot 76:598–602. doi:10.1038/s41429-023-00639-6 37402884

[13] Lu Y, Li MC, Liu HC, Lin SQ, Zhao XQ, Liu ZG, Zhao LL, Wan KL. 2021. Detecting Mycobacterium tuberculosis complex and rifampicin resistance via a new rapid multienzyme isothermal point mutation assay. Anal Biochem 630:114341. doi:10.1016/j.ab.2021.114341 34411551

[14] Sun M-L, Lai H-Y, Chong N-Y, Liu D-F, Zhang Z-Y, Pang B, Yao J. 2021. Simple and feasible detection of hepatitis B virus via combination of multienzyme isothermal rapid amplification and lateral flow dipstick strip. Front Mol Biosci 8:763079. doi:10.3389/fmolb.2021.763079 34926579 PMC8674754

[15] Wang X, Shang X, Huang X. 2020. Next-generation pathogen diagnosis with CRISPR/Cas-based detection methods. Emerg Microbes Infect 9:1682–1691. doi:10.1080/22221751.2020.1793689 32643563 PMC7473117

[16] Huang W, Zhang Z, Lin D, Deng Y, Chen X, Huang J. 2023. RT-nestRPA is a new technology for the rapid and sensitive detection of nucleic acid detection of pathogens used for a variety of medical application scenarios. Anal Chim Acta 1262:341263. doi:10.1016/j.aca.2023.341263 37179064 PMC10123537

[17] Zhang Y, Hai Y, Duan B, Long H, Xie X, Teng Z, Yin F, Wang M, Xiong Y, Shao Z, Guo W, Qin A. 2023. A seminested recombinase polymerase amplification assay to detect rickettsial pathogens in clinical samples. Diagn Microbiol Infect Dis 107:116067. doi:10.1016/j.diagmicrobio.2023.116067 37751629

[18] Association C. 2005. China clinical treatment guide for tuberculosis

[19] World Health Organization. 2010. Treatment of tuberculosis: guidelines, 4th Ed. World Health Organization, Geneva.

[20] Luetkemeyer AF, Firnhaber C, Kendall MA, Wu X, Mazurek GH, Benator DA, Arduino R, Fernandez M, Guy E, Johnson P, Metchock B, Sattler F, Telzak E, Wang YF, Weiner M, Swindells S, Sanne IM, Havlir DV, Grinsztejn B, Alland D, AIDS Clinical Trials Group A5295 and Tuberculosis Trials Consortium Study 34 Teams. 2016. Evaluation of Xpert MTB/RIF versus AFB smear and culture to identify pulmonary tuberculosis in patients with suspected tuberculosis from low and higher prevalence settings. Clin Infect Dis 62:1081–1088. doi:10.1093/cid/ciw035 26839383 PMC4826450

[21] Asmar S, Chatellier S, Mirande C, van Belkum A, Canard I, Raoult D, Drancourt M. 2016. A chlorhexidine-agar plate culture medium protocol to complement standard broth culture of Mycobacterium tuberculosis. Front Microbiol 7:30. doi:10.3389/fmicb.2016.00030 26834733 PMC4725127

[22] Pai M, Dewan PK, Swaminathan S. 2023. Transforming tuberculosis diagnosis. Nat Microbiol 8:756–759. doi:10.1038/s41564-023-01365-3 37127703

[23] Singpanomchai N, Akeda Y, Tomono K, Tamaru A, Santanirand P, Ratthawongjirakul P. 2021. Rapid detection of multidrug-resistant tuberculosis based on allele-specific recombinase polymerase amplification and colorimetric detection. PLoS ONE 16:e0253235. doi:10.1371/journal.pone.0253235 34115793 PMC8195408

[24] Singpanomchai N, Akeda Y, Tomono K, Tamaru A, Santanirand P, Ratthawongjirakul P. 2019. Naked eye detection of the Mycobacterium tuberculosis complex by recombinase polymerase amplification-SYBR green I assays. J Clin Lab Anal 33:e22655. doi:10.1002/jcla.22655 30129085 PMC6818612

[25] Liu D, Zhao B, Ou X, Zheng H, Ma A, He W, Wang S, Zhou Y, Xia H, Zheng Y, Hou P, He G, Zhao Y. 2018. A novel isothermal amplification-based method to detect Mycobacterium tuberculosis complex. J Microbiol Methods 145:59–65. doi:10.1016/j.mimet.2017.11.003 29109010

[26] Marlowe EM, Novak-Weekley SM, Cumpio J, Sharp SE, Momeny MA, Babst A, Carlson JS, Kawamura M, Pandori M. 2011. Evaluation of the Cepheid Xpert MTB/RIF assay for direct detection of Mycobacterium tuberculosis complex in respiratory specimens. J Clin Microbiol 49:1621–1623. doi:10.1128/JCM.02214-10 21289151 PMC3122817

[27] Helb D, Jones M, Story E, Boehme C, Wallace E, Ho K, Kop J, Owens MR, Rodgers R, Banada P, Safi H, Blakemore R, Lan NTN, Jones-López EC, Levi M, Burday M, Ayakaka I, Mugerwa RD, McMillan B, Winn-Deen E, Christel L, Dailey P, Perkins MD, Persing DH, Alland D. 2010. Rapid detection of Mycobacterium tuberculosis and rifampin resistance by use of on-demand, near-patient technology. J Clin Microbiol 48:229–237. doi:10.1128/JCM.01463-09 19864480 PMC2812290

[28] Blakemore R, Story E, Helb D, Kop J, Banada P, Owens MR, Chakravorty S, Jones M, Alland D. 2010. Evaluation of the analytical performance of the Xpert MTB/RIF assay. J Clin Microbiol 48:2495–2501. doi:10.1128/JCM.00128-10 20504986 PMC2897495

[29] Chakravorty S, Simmons AM, Rowneki M, Parmar H, Cao Y, Ryan J, Banada PP, Deshpande S, Shenai S, Gall A, Glass J, Krieswirth B, Schumacher SG, Nabeta P, Tukvadze N, Rodrigues C, Skrahina A, Tagliani E, Cirillo DM, Davidow A, Denkinger CM, Persing D, Kwiatkowski R, Jones M, Alland D. 2017. The new Xpert MTB/RIF ultra: improving detection of Mycobacterium tuberculosis and resistance to rifampin in an assay suitable for point-of-care testing. MBio 8:e00812-17. doi:10.1128/mBio.00812-17 28851844 PMC5574709

[30] Zein-Eddine R, Refrégier G, Cervantes J, Yokobori NK. 2023. The future of CRISPR in Mycobacterium tuberculosis infection. J Biomed Sci 30:34. doi:10.1186/s12929-023-00932-4 37245014 PMC10221753

[31] Xiao J, Li J, Quan S, Wang Y, Jiang G, Wang Y, Huang H, Jiao W, Shen A. 2023. Development and preliminary assessment of a CRISPR-Cas12a-based multiplex detection of Mycobacterium tuberculosis complex. Front Bioeng Biotechnol 11:1233353. doi:10.3389/fbioe.2023.1233353 37711452 PMC10497956

[32] Dorman SE, Schumacher SG, Alland D, Nabeta P, Armstrong DT, King B, Hall SL, Chakravorty S, Cirillo DM, Tukvadze N, et al.. 2018. Xpert MTB/RIF Ultra for detection of Mycobacterium tuberculosis and rifampicin resistance: a prospective multicentre diagnostic accuracy study. Lancet Infect Dis 18:76–84. doi:10.1016/S1473-3099(17)30691-6 29198911 PMC6168783

[33] Ai JW, Zhou X, Xu T, Yang M, Chen Y, He GQ, Pan N, Cai Y, Li Y, Wang X, Su H, Wang T, Zeng W, Zhang WH. 2019. CRISPR-based rapid and ultra-sensitive diagnostic test for Mycobacterium tuberculosis Emerg Microbes Infect 8:1361–1369. doi:10.1080/22221751.2019.1664939 31522608 PMC6758691

[34] Sam IK, Chen YY, Ma J, Li SY, Ying RY, Li LX, Ji P, Wang SJ, Xu J, Bao YJ, Zhao GP, Zheng HJ, Wang J, Sha W, Wang Y. 2021. TB-QUICK: CRISPR-Cas12b-assisted rapid and sensitive detection of Mycobacterium tuberculosis. J Infect 83:54–60. doi:10.1016/j.jinf.2021.04.032 33951419

[35] Ren W, Zhou Y, Li H, Shang Y, Zhang X, Yuan J, Li S, Li C, Pang Y. 2023. Development and clinical evaluation of a CRISPR/Cas13a-based diagnostic test to detect Mycobacterium tuberculosis in clinical specimens. Front Microbiol 14:1117085. doi:10.3389/fmicb.2023.1117085 36819015 PMC9935578

[36] Xu H, Zhang X, Cai Z, Dong X, Chen G, Li Z, Qiu L, He L, Liang B, Liu X, Liu J. 2020. An isothermal method for sensitive detection of Mycobacterium tuberculosis complex using clustered regularly interspaced short palindromic repeats/Cas12a Cis and trans cleavage. J Mol Diagn 22:1020–1029. doi:10.1016/j.jmoldx.2020.04.212 32470556

[37] Jiang H, Li Y, Lv X, Deng Y, Li X. 2023. Recent advances in cascade isothermal amplification techniques for ultra-sensitive nucleic acid detection. Talanta 260:124645. doi:10.1016/j.talanta.2023.124645 37148686 PMC10156408

[38] Kang Y, Wang J, Zhang W, Xu Y, Xu B, Qu G, Yu Y, Yan B, Su G. 2023. RNA extraction-free workflow integrated with a single-tube CRISPR-Cas-based colorimetric assay for rapid SARS-CoV-2 detection in different environmental matrices. J Hazard Mater 454:131487. doi:10.1016/j.jhazmat.2023.131487 37148798 PMC10125216

[39] Huang Z, Zhang G, Lyon CJ, Hu TY, Lu S. 2023. Outlook for CRISPR-based tuberculosis assays now in their infancy. Front Immunol 14:1172035. doi:10.3389/fimmu.2023.1172035 37600797 PMC10436990

