# Supplementary material for: Rapid and sensitive detection of Mycobacterium tuberculosis using nested multi-enzyme isothermal rapid amplification in a single reaction
Source: Microbiol Spectr. 2024 Oct 28;12(12):e00887-24. doi: 10.1128/spectrum.00887-24 (PMC11619386; doi:10.1128/spectrum.00887-24)
Supplement: Supplemental figures — Fig. S1 and S2. [file spectrum.00887-24-s0001.docx]

**Supplementary Figures**


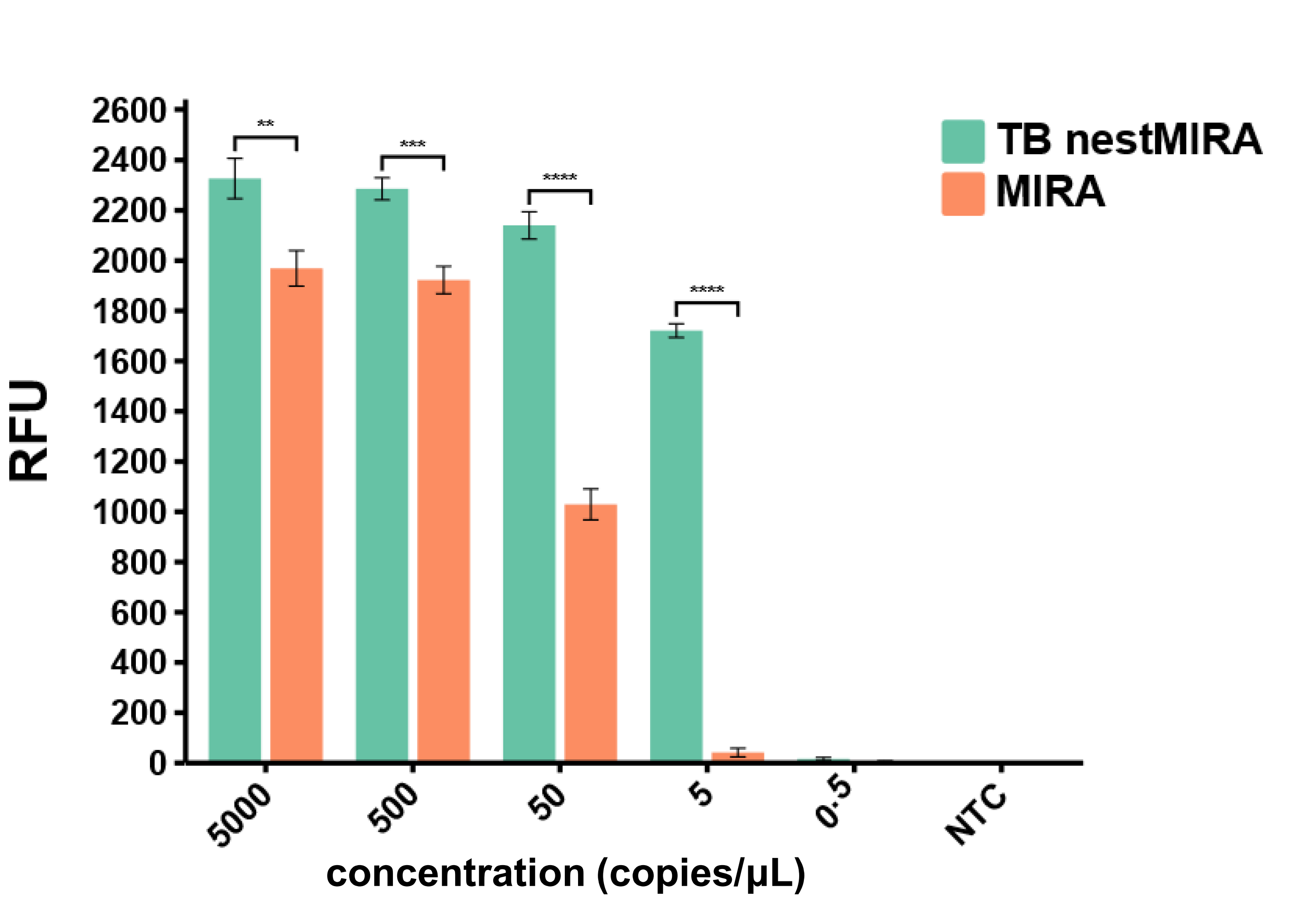


FIG S1. Comparison of results of TB nestMIRA and MIRA for different concentrations of Mtb DNA detection


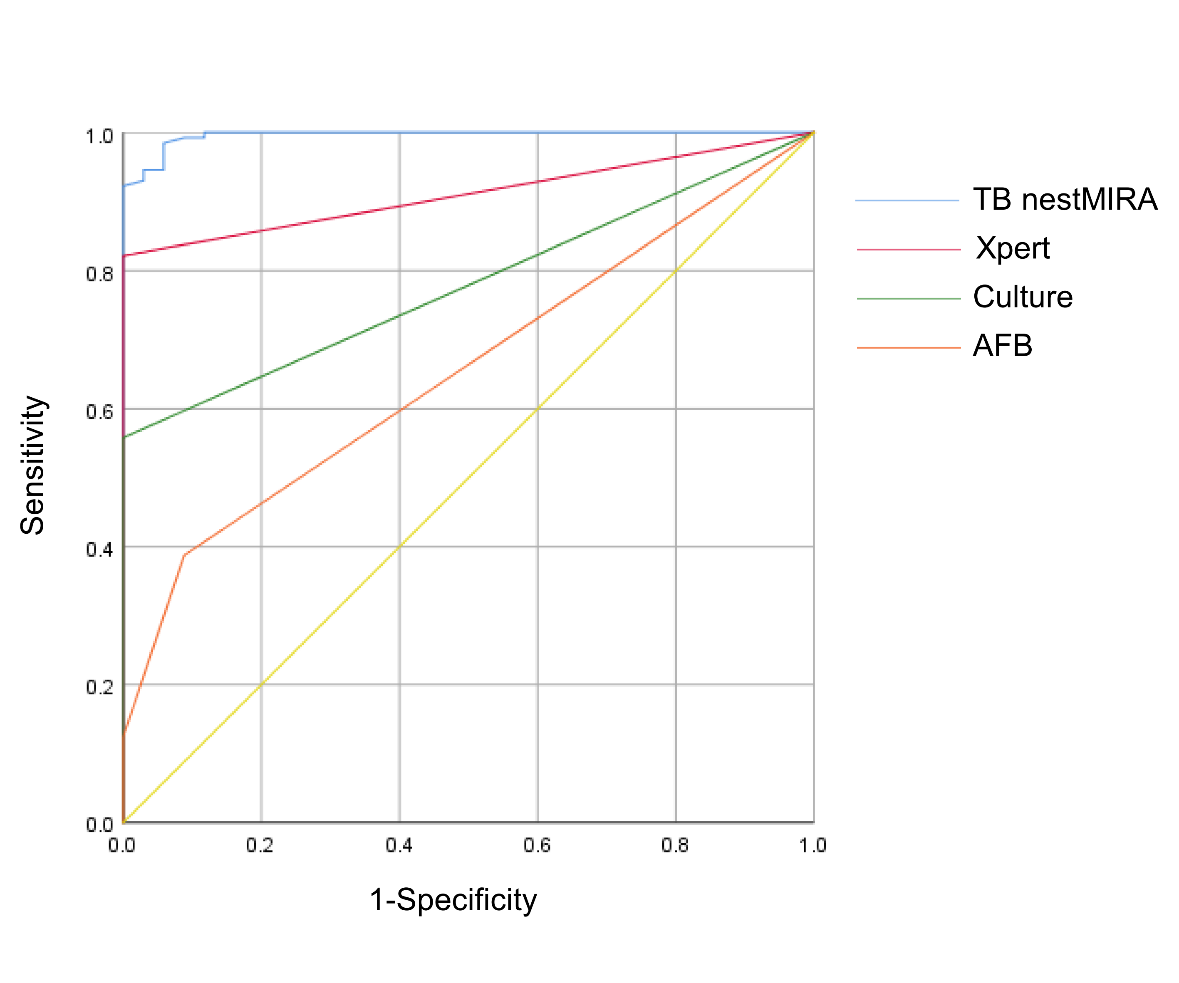


FIG S2. Receiver operating characteristic curve for four TB detection methods, including TB nestMIRA, Xpert, Cluture, and AFB smear
