# Supplementary material for: Rapid and sensitive detection of Mycobacterium tuberculosis using nested multi-enzyme isothermal rapid amplification in a single reaction
Source: Microbiol Spectr. 2024 Oct 28;12(12):e00887-24. doi: 10.1128/spectrum.00887-24 (PMC11619386; doi:10.1128/spectrum.00887-24)
Supplement: Supplemental tables — Tables S1 to S3. [file spectrum.00887-24-s0002.docx]

**Supplementary Tables**

TABLE S1 The primers and probes used in the study

| Primer name | | Sequence 5′–3′ | Length |
| --- | --- | --- | --- |
| Inner primer | 1NF | GATCCTGCGAGCGTAGGCGTCGGTGACAAAGGCC | 34nt |
|  | 2NF | CAGGATCCTGCGAGCGTAGGCGTCGGTGAC | 30nt |
|  | 3NF | TGGAGGTGGCCATCGTGGAAGCGACCCGCCAG | 32nt |
|  | 1NR | GCAGGACCACGATCGCTGATCCGGCCACAG | 30nt |
|  | 2NR | TCCCGCCGATCTCGTCCAGCGCCGCTTCGG | 31nt |
|  | 3NR | CGCAGGACCACGATCGCTGATCCGGCCACA | 30nt |
| Outer primer | 1WF | CTTTCAGGTCGAGTACGCCTTCTTGTTGGC | 30nt |
|  | 2WF | CGGGTCCAGATGGCTTGCTCGATCGCGTCG | 30nt |
|  | 3WF | TGCTCGATCGCGTCGAGGACCATGGAGGTG | 30nt |
|  | 1WR | TGACCAAACTCGGCCTGTCCGGGACCACCCG | 31nt |
|  | 2WR | CGCAAAGTGTGGCTAACCCTGAACCGTGAG | 30nt |
|  | 3WR | CCCGCAAAGTGTGGCTAACCCTGAACCGTG | 30nt |
| Probe | IS6110-F-P | GAACCCTGCCCAGGTCGACACATAGGTGAGGTC (dTFAM)G/idSp/(dTBHQ1) ACCCACAGCCGGT  TA-Spacer C3 | 53nt |
|  | IS6110-LFA-P | FAM-GAACCCTGCCCAGGTCGACACATAGGTGAGGTCTG/idSp/TACCCACAGCCGGTTA-Spacer C3 | 53nt |

NCBI Reference Sequence: NC_000962.3

TABLE S2 Demographic and clinical characteristics of the study participants

| Characteristics | TB patient  n=129 | Non-TB patient  n=34 |
| --- | --- | --- |
| Age，median(range) | 42(15-86) | 43(12-83) |
| <18 | 8(6.2%) | 3(8.8%) |
| 18-60 | 106(82.2%) | 27(79.4%) |
| >60 | 15(11.6%) | 4(11.7%) |
| Male | 83(64.3%) | 23(67.6%) |
| Treatment status |  |  |
| New case | 118(91.5%) | / |
| Retreated case | 11(8.5%) | / |
| Pulmonary | 112(86.8%) | / |
| Extrapulmonary | 17(13.2%) | / |
| Blood-borne TB | 3(17.6%) | / |
| Urinary tract TB | 6(35.3%） | / |
| TB Pleurisuy | 8(47.1%) | / |
| HIV-negative | 129(100%) | 34(100%) |

TABLE S3 Number of positive detections of tuberculosis patients by different testing methods

| **Positive for only one tests** | | | | | | |
| --- | --- | --- | --- | --- | --- | --- |
|  | TB nestMIRA | Xpert | Culture | AFB |  |  |
| Number of positives | 7 | 6 | 1 | 1 |  |  |
| **Positive for any two tests** | | | | | | |
|  | TB nestMIRA  & Xpert | TB nestMIRA  & Culture | TB nestMIRA  & AFB | Xpert  & Culture | Xpert  & AFB | Culture  & AFB |
| Number of positives | 29 | 2 | 1 | 0 | 0 | 0 |
| **Positive for any Three tests** | | | | | | |
|  | TB nestMIRA  & Xpert  & Culture | TB nestMIRA  & Xpert  & AFB | TB nestMIRA  & Culture  & AFB | Xpret  & Culture  & AFB |  |  |
| Number of positives | 22 | 2 | 0 | 0 |  |  |
| **Positive for all tests** | | | | | | |
|  | TB nestMIRA & Xpert  & Culture & AFB | |  |  |  |  |
| Number of positives | 47 | |  |  |  |  |
